# Tlx3 Promotes Glutamatergic Neuronal Subtype Specification through Direct Interactions with the Chromatin Modifier CBP

**DOI:** 10.1371/journal.pone.0135060

**Published:** 2015-08-10

**Authors:** Atsushi Shimomura, Dharmeshkumar Patel, Sarah M. Wilson, Karl R. Koehler, Rajesh Khanna, Eri Hashino

**Affiliations:** 1 Stark Neurosciences Research Institute, Indiana University School of Medicine, Indianapolis, Indiana, United States of America; 2 Department of Otolaryngology-Head and Neck Surgery, Indiana University School of Medicine, Indianapolis, Indiana, United States of America; 3 Department of Pharmacology and Toxicology, Indiana University School of Medicine, Indianapolis, Indiana, United States of America; 4 School of Psychological Science, Health Sciences University of Hokkaido, Sapporo, Hokkaido, Japan; 5 Department of Pharmacology, University of Arizona School of Medicine, Tucson, Arizona, United States of America; University of Wurzburg, GERMANY

## Abstract

Nervous system development relies on the generation of precise numbers of excitatory and inhibitory neurons. The homeodomain transcription factor, T-cell leukemia 3 (Tlx3), functions as the master neuronal fate regulator by instructively promoting the specification of glutamatergic excitatory neurons and suppressing the specification of gamma-aminobutyric acid (GABAergic) neurons. However, how Tlx3 promotes glutamatergic neuronal subtype specification is poorly understood. In this study, we found that Tlx3 directly interacts with the epigenetic co-activator cyclic adenosine monophosphate (cAMP)-response element-binding protein (CREB)-binding protein (CBP) and that the Tlx3 homeodomain is essential for this interaction. The interaction between Tlx3 and CBP was enhanced by the three amino acid loop extension (TALE)-class homeodomain transcription factor, pre-B-cell leukemia transcription factor 3 (Pbx3). Using mouse embryonic stem (ES) cells stably expressing Tlx3, we found that the interaction between Tlx3 and CBP became detectable only after these Tlx3-expressing ES cells were committed to a neural lineage, which coincided with increased Pbx3 expression during neural differentiation from ES cells. Forced expression of mutated Tlx3 lacking the homeodomain in ES cells undergoing neural differentiation resulted in significantly reduced expression of glutamatergic neuronal subtype markers, but had little effect on the expression on pan neural markers. Collectively, our results strongly suggest that functional interplay between Tlx3 and CBP plays a critical role in neuronal subtype specification, providing novel insights into the epigenetic regulatory mechanism that modulates the transcriptional efficacy of a selective set of neuronal subtype-specific genes during differentiation.

## Introduction

In the vertebrate nervous system, neurons can be classified as excitatory glutamatergic or inhibitory gamma-aminobutyric acid (GABAergic) neurons. Precise control over the generation of these two primary neuronal subtypes allows the formation of appropriate neural networks, thereby facilitating higher nervous system functions. An imbalance between glutamatergic and GABAergic neurons is frequently associated with nervous system disorders such as hyperalgesia, epilepsy, and mental retardation [1, 2]. Thus, a clear understanding of the molecular mechanisms that govern fate choices between glutamatergic and GABAergic neurons not only has scientific importance, but is also critical for elucidating the etiology of various neurological disorders.

The transcription factor, T-cell leukemia 3 (Tlx3; also known as Hox11-L2/Rnx), is a member of the Tlx/Hox11 subfamily of Hox homeodomain transcription factors, which are expressed in several developing neural tissues such as the hindbrain, cranial sensory ganglia, dorsal root ganglia, and dorsal spinal cord [3, 4]. Tlx3-deficient mice exhibit aberrant development of somatic sensory cells in the dorsal horn of the spinal cord and abnormalities in the formation of first-order relay visceral sensory neurons in the brainstem [5–7]. Ectopic Tlx3 expression in the developing chick neural tube is sufficient to suppress GABAergic cell differentiation and to induce the generation of glutamatergic neurons [6], indicating that the Tlx3 protein serves as a “selector” that promotes the glutamatergic neural fate over the GABAergic neural fate. Consistent with this, Tlx3 is responsible for controlling the expression of transmitter transporter and receptor genes associated with GABAergic and glutamatergic neurons in the developing dorsal spinal cord [8]. Despite the established role for Tlx3 in glutamatergic neuronal subtype specification, little is known about the mechanisms underlying Tlx3-mediated target gene transcription.

Previous studies have shown that the choice between the glutamatergic and GABAergic neuronal subtypes is controlled by complex transcription factor regulatory networks [9–11]. Rather than functioning as monomers, transcription factors often form protein complexes by recruiting various transcriptional cofactors [12–14]. These cofactors function as epigenetic regulators that alter chromatin structure [15–17], thereby modulating the efficiency of gene transcription. Accordingly, epigenetic regulatory factors comprise an essential part of the transcriptional regulatory mechanisms that control the proper expression of neuronal subtype-determinant genes. Recent genome-wide analyses have supported this hypothesis by demonstrating the involvement of various epigenetic regulators in neuronal subtype specification, including genes that mediate DNA methylation, histone modifications, and chromatin remodeling enzymes [18]. One of these epigenetic cofactors is the cyclic adenosine monophosphate (cAMP)-response element-binding protein (CREB)-binding protein (CBP). CBP is a transcriptional co-activator that controls transcription via direct interactions with transcription factors and the basal transcription machinery as well as its histone acetyltransferase (HAT) activity, which transforms chromatin into a more relaxed structure to enable the transcription of target genes [19–21]. Because CBP has been shown to interact with various transcription factors [22, 23], we hypothesized that Tlx3 and CBP cooperatively mediate glutamatergic neuronal cell fate specification through their direct interactions. To test this hypothesis, we employed a well-characterized *in vitro* system where Tlx3 promotes context-dependent glutamatergic neuronal specification from mouse embryonic stem (ES) cells [4].

## Materials and Methods

### Animal experiments and ethics statement

Pregnant mice were euthanized by CO_2_ inhalation, and the embryos were surgically removed by trained personnel. All animal experiments were performed in strict accordance with protocols approved by the Indiana University School of Medicine Institutional Animal Care and Use Committee.

### DNA constructs

To facilitate the identification and selection of cells transfected with the pBud-CE4.1 vector, pBud-enhanced green fluorescent protein (pBud-eGFP) cDNA was inserted between the *Kpn*I and *Xho*I sites of the pBud-CE4.1 vector to allow eGFP expression under the control of the elongation factor 1α promoter, as described earlier [4]. To create a wild-type Tlx3 expression vector with the myc epitope tag at the Tlx3 carboxyl terminus (pBud-eGFP-Tlx3-myc), full-length mouse Tlx3 cDNA was amplified by polymerase chain reaction (PCR) using the FANTOM clone (AK141870; RIKEN Brain Science Institute, Japan) and appropriate primer sets (Tlx3-F and Tlx3-R, Table 1), before it was subcloned between *Xba*I and *Bam*HI in-frame with the myc epitope to allow expression under the control of the pBud-eGFP cytomegalovirus (CMV) promoter. To obtain a C-terminal deletion mutant (Tlx3ΔC) expression vector (pBud-eGFP-Tlx3ΔC-myc), Tlx3ΔC was PCR amplified using the Tlx3ΔC-F and Tlx3ΔC-R primers corresponding to amino acids 1–226 (Table 1) and inserted into the pBud-eGFP vector in-frame with the myc epitope tag to allow expression under the control of the CMV promoter. The expression vector coding the homeodomain deletion mutant of Tlx3 (pBud-eGFP-Tlx3ΔHD-myc) was generated by inverse PCR using Tlx3ΔHD-F and Tlx3ΔHD-R primers (Table 1) that allowed the deletion of amino acids 166–226, which was followed by *Dpn*I digestion of pBud-eGFP-Tlx3-myc and blunt-end ligation of Tlx3ΔHD-myc. A Tlx3 construct that harbored a point mutation from phenylalanine to glycine at position 127 (Tlx3W127G) in the Hox11 Pbx interaction motif was introduced into the pBud-eGFP-Tlx3-myc vector using a QuikChange site-directed mutagenesis kit (Stratagene, Santa Clara, CA, USA) according to the manufacturer’s instructions using the primer sets Tlx3W127G-F and Tlx3W127G-R (Table 1). To produce a Pbx3 expression construct, the full-length mouse Pbx3 cDNA was amplified from a mouse ES cell cDNA library using Pbx3-F and Pbx3-R (Table 1) and then inserted between the *Bam*HI and *Kpn*I sites in the p3xFLAG-CMV-10 expression vector, which encodes three adjacent Flag epitopes upstream of the inserted *Pbx3* gene. The construct was verified by sequencing. A full-length CBP expression vector, pRc/RSV-CBP, was purchased from Origene (Rockville, MD, USA).

**Table 1:** List of oligonucleotide primers used for vector construction.

Name	Sequences (5’>3’)
Tlx3-F	GCTCTAGAATGGAGGCGCCCGCCAG
Tlx3-R	CGGGATCCCACCAGAGAGGTGACAGC
Tlx3-ΔC-F	GCTCTAGAATGGAGGCGCCCGCCAG
Tlx3-ΔC-R	CGGGATCCCGCCGTCTGCCGCCTCCACTTGG
Tlx3-ΔHD-F	GCGGAGGAGCGGGAGGCGGAC
Tlx3-ΔHD-R	CTTGGGTGGCGTCCGGTTCTGG
Tlx3W127G-F	GGCCTCAATTTCCCCGGGATGGAGAGCAGTC
Tlx3W127G-R	GACTGCTCTCCATCCCGGGGAAATTGAGGCC
Pbx3-F	CGGGTACCGATGGACGATCAATCCAGGATGC
Pbx3-R	GCGGATCCTTAGTTAGAGGTATCCGAGTGCAC

F, forward; R, reverse.

### Cell culture and transfection

HEK 293 cells were maintained in Dulbecco’s modified Eagle’s medium (DMEM) supplemented with 10% fetal bovine serum (FBS) and 0.01% penicillin/streptomycin (Stemcell Technologies, Vancouver, BC, Canada) at 37°C in a 5% CO_2_ atmosphere. Transfections were performed using Lipofectamine 2000 reagent (Life Technologies; Carlsbad, CA, USA) according to the manufacturer’s instructions. The total amounts of transfected DNA were adjusted with the empty p3xFLAG-CMV-10. The transfected cells were then cultured for 40 h before harvesting for immunoprecipitation.

Undifferentiated R1 mouse ES cells were grown on gelatin-coated culture dishes in high-glucose DMEM supplemented with 15% FBS (Life Technologies), 1.0 mM sodium pyruvate (Stemcell Technologies), 10 mM nonessential amino acids (Stemcell Technologies), 0.01% penicillin/streptomycin (Stemcell Technologies), 2.0 mM l-glutamine (Stemcell Technologies), 1000 units/ml leukemia inhibiting factor (Merck Millipore, Darmstadt, Germany), and 0.055 mM 2-mercaptoethanol. Transfections were performed using a mouse ES cell nucleofection kit (VPH-1001; Lonza) and an Amaxa Nucleofector device (Lonza, Basel, Switzerland). After transfection, the cells were incubated in growth medium containing 50 g/ml Zeosin (Life Technologies) to select stable transfectants.

### Neural differentiation of ES cells

The neural differentiation of ES cells was induced according to our previously established protocol [4]. Briefly, stably transfected ES cells were dissociated in 0.25% trypsin–ethylenediaminetetraacetic acid (EDTA) and cultured on bacterial-grade dishes in differentiation medium containing G-MEM (Life Technologies), 5% knockout serum replacement (Life Technologies), 2.0 mM l-glutamine, 1.0 mM sodium pyruvate, 0.1 mM nonessential amino acids, 0.01% penicillin/streptomycin, and 0.1 mM 2-mercaptoethanol to allow the formation of embryoid bodies (EBs). After 5 days, EBs were transferred into tissue culture plates or chamber slides, which were double coated with poly-d-lysine (200 g/ml) and mouse laminin (10 g/ml) and then cultured for another 2 days in differentiation medium. Next, the medium was replaced with neural induction medium containing G-MEM, 1% N2, 2 mM glutamine, 1 mM pyruvate, 0.1 mM nonessential amino acids, 0.1 mM 2-mercaptoethanol, 0.01% penicillin/streptomycin, and 10 ng/ml brain-derived neurotrophic factor (PeproTech, Rocky Hill, NJ, USA). The day when the medium was changed from differentiation to neural induction medium was defined as neural induction day 0. The cells were maintained in neural induction medium for 4–7 days.

### Cell extracts, immunoprecipitation, and immunoblotting

To prepare whole-cell lysates for immunoprecipitation, the transfected HEK293 cells were lysed by sonication in immunoprecipitation buffer containing 10 mM Tris–HCl, pH 7.5, 150 mM NaCl, 1 mM EDTA, 12% glycerol, 1% Nonidet P-40, 1 mM dithiothreitol (DTT), protease inhibitor mixture, and phosphatase inhibitor mixture. The soluble fraction was isolated by centrifugation at 15,000 rpm for 10 min and then diluted in immunoprecipitation buffer without NaCl and glycerol to achieve a final concentration of 75 mM NaCl and 6% glycerol in the immunoprecipitation reaction mixtures.

The nuclear proteins in ES cell-derived neurons and embryonic tissues of ICR mice were extracted as described previously [4, 24], with some modifications. Briefly, cells or tissues were suspended in Dignam buffer A [10 mM 4-(2-hydroxyethyl)-1-piperazineethanesulfonic acid (HEPES), pH 7.9, 10 mM KCl, 0.1 mM EDTA, 1 mM DTT, protease inhibitor mixture, and phosphatase inhibitor mixture], kept on ice for 15 min, and subsequently extracted in 0.6% Nonidet P-40. The extract was vortexed and centrifuged for 3 min at 500 × *g* to pellet the nuclei. The pellet was resuspended in Dignam buffer C (20 mM HEPES, 1.5 mM MgCl_2_, 420 mM NaCl, 0.2 mM EDTA, 25% glycerol, 1 mM DTT, protease inhibitor, and phosphatase inhibitors), incubated for 1 h with shaking to extract nuclear proteins, and then centrifuged for 15 min at 18,000×*g*. Immunoprecipitation buffer was used for the final dialysis step. The dialysate was subsequently centrifuged for 15 min at 18,000×*g*, and the supernatant used as the nuclear extract. To extract the total protein from ES cells, the cells were lysed in radioimmunoprecipitation assay (RIPA) buffer (10 mM Tris–HCl, pH 7.4, 150 mM NaCl, 1 mM EDTA, 1% Nonidet P-40, 0.1% sodium deoxycholate, 0.1% sodium dodecyl sulfate (SDS), protease inhibitor mixture, and phosphatase inhibitor mixture). The lysates were cleared by centrifugation at 20,000 × g for 10 min in a microcentrifuge.

Before immunoprecipitation, the lysates were precleared with protein G-Sepharose beads, and they were then incubated with one of the following antibodies: anti-Myc (Santa Cruz Biotechnology, Dallas, TX, USA), anti-CBP (Abcam, Cambridge, MA, USA), or anti-Tlx3 (Santa Cruz Biotechnology). The cell lysate and antibody mixture was incubated overnight at 4°C. The following day, protein G-Sepharose beads were added and incubated for 2 h. The beads were harvested by gentle centrifugation and washed three times with a wash buffer containing 10 mM Tris-HCl, pH 7.5, 75 mM NaCl, 1 mM EDTA, 6% glycerol, 1% NP-40, 1 mM DTT, protease inhibitor mixture, and phosphatase inhibitor mixture.

For the immunoblot analyses, immunoprecipitated samples or cell extracts were resuspended in Laemmli sample buffer and resolved by SDS-polyacrylamide gel electrophoresis on 4%–20% gradient gels and then transferred to Hybond-P/PVDF membranes. The membranes were blocked in phosphate-buffered saline (PBS) containing 1% nonfat milk and 0.05% Tween-20 for 1 h, followed by incubation overnight at 4°C with primary antibodies (anti-Myc, anti-CBP, anti-FLAG, and anti-Tlx3). The membranes were then washed three times with PBS containing 0.05% Tween-20 and incubated for 1 h with horseradish peroxidase-conjugated secondary antibodies. Immunoreactive protein bands were visualized using the SuperSignal West Pico or Femto chemiluminescent detection systems (Thermo Fisher Scientific, Waltham, MA, USA), following by exposure to X-ray film for appropriate durations to ensure that the signals were not saturated. The films were then scanned and digitized.

### Quantitative RT-PCR

Total RNA was extracted from cells using an RNeasy Mini kit (Qiagen, Hilden, Germany) according to the manufacturer’s protocol. All of the RNA samples were first treated with Ambion Turbo DNase (Life Technologies) to remove genomic DNA contamination and then reverse transcribed into cDNAs using oligodT primers and Omniscript reverse transcriptase (Qiagen). Quantitative RT-PCR analyses were performed as described previously [25, 26]. The primer sequences used are listed in Table 2.

**Table 2:** List of oligonucleotide primers used for quantitative RT-PCR.

Name	Sequences (5’>3’)
L27-F	ACAACCACCTCATGCCCACA
L27-R	CTGGCCTTGCGCTTCAAA
Pbx3-F	CCTTGGAGCAAACTCACTGT
Pbx3-R	GCCTTCCGTAGGAGAAGTCA
NeuroD-F	CCTGATCTGGTCTCCTTCGT
NeuroD-R	AAGAAAGTCCGAGGGTTGAG
Ngn1-F	TCGGCTTCAGAAGACTTCAC
Ngn1-R	GTGGTATGGGATGAAACAGG
GluR2-F	CTGGTGGCTTTGATTGAGTT
GluR2-R	AATTCTGCGAGGAAGATGG
GluR4-F	AAGCAAGGACAAGACGAGTG
GluR4-R	CTATCAAAGCCACCAGCATT
Vglut2-F	ACCCAGATTCCAGGAGGATA
Vglut2-R	GCAGATGGGATCAGCATATT
Tau-F	TAGCAACGTCCAGTCCAAGT
Tau-R	GTCACTTTGCTCAGGTCCAC

F, forward; R, reverse.

### Immunofluorescence staining

Cells cultured on chamber slides were fixed with 4% paraformaldehyde. The fixed cells were then blocked with blocking solution and incubated with a mixture of rabbit polyclonal anti-GluR4 (Millipore) and mouse monoclonal anti-TUJ1 (Covance, Princeton, NJ, USA). Immunoreactivity with polyclonal and monoclonal antibodies was visualized using AlexaFluor 568-conjugated anti-rabbit IgG and AlexaFluor 488-conjugated anti-mouse IgG, respectively. Images of stained cells were acquired using a Nikon Eclipse TE2000-U inverted microscope (Nikon, Tokyo, Japan) at 20× or 40× magnification. The immunohistochemical analysis of mouse embryos was performed as described previously [4].

### Flow Cytometry

Flow cytometry was conducted as described previously [4]. Antibodies against Tuj1 (Covance) and GluR2/3 (Millipore) were conjugated to Alexa Fluor 647 using an antibody labeling kit (Thermo Fisher Scientific). The cells were fixed as described, permeabilized with 0.1% Triton X-100, and incubated overnight with fluorophore-conjugated anti-Tuji1 or anti-GluR2/3. The cells were sorted using a FACSCalibur flow cytometry system (BD Biosciences, Franklin Lakes, NJ, USA).

### Fura-2 calcium imaging

Fura-2 microfluorometry was performed as described previously [27]. Neurally differentiated ES cells were loaded for 30 min at 37°C with 3 μM fura-2 acetoxymethyl ester (a Ca^2+^-sensitive fluorescent dye) in Tyrode’s buffer (119 mM NaCl, 2.5 mM KCl, 2 mM CaCl_2_, 2 mM MgCl_2_, 25 mM HEPES, pH 7.4, and 30 mM glucose). The fluorescence ratio was measured throughout the entire cell using a digital video microfluorometry system, which comprised an intensified charge-coupled device camera coupled to a microscope controlled by Nikon Elements software. The excitation wavelength was alternated between 340 and 380 nm, and fluorescent emissions were monitored at 510 nm. After obtaining the baseline fluorescent emissions, the cells were stimulated with 100 μM glutamate in Tyrode’s buffer.

### Statistical analysis

Statistical comparisons were performed using a one-way ANOVA with post-hoc Tukey’s tests or paired *t*-tests, as appropriate. The level of significance was set at *p* < 0.05.

## Results

### Tlx3 interacts with CBP through its homeodomain

**Fig 1:**
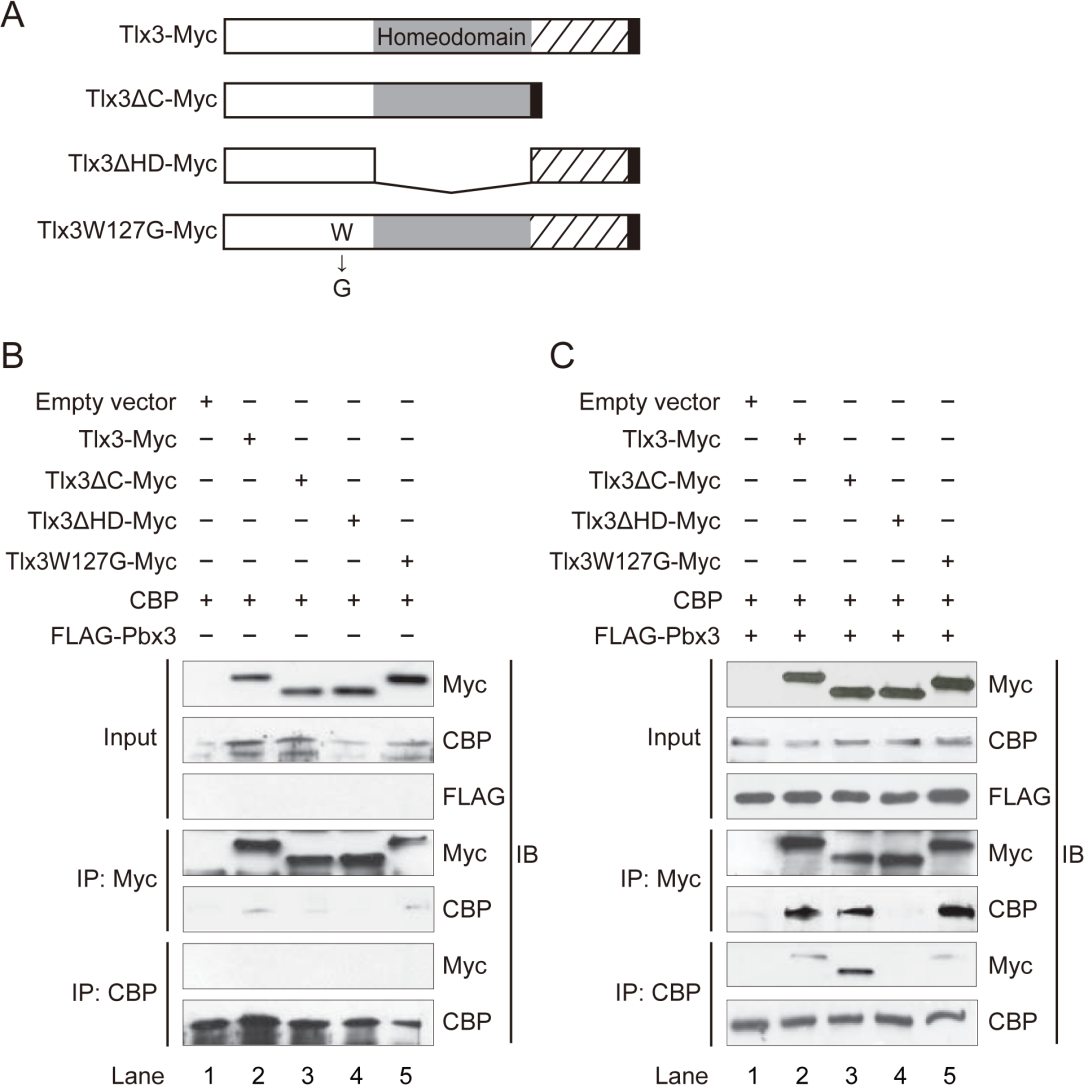
Tlx3 interacts with CBP through the homeodomain. (**A**) Schematic representation of the wild-type Tlx3 protein and the deletion and point mutants used in this study. The oblique lines delineate the Tlx3-specific (compared with other Tlx/Hox11 subfamily members) carboxyl-terminal region. The gray box denotes the putative homeodomain. The Myc tag is represented by the solid black box. The Tlx3ΔC and Tlx3ΔHD mutants lack the carboxyl-terminal region (amino acids 227–291) and the homeodomain (amino acids 166–226), respectively. The Tlx3-W127G mutant carries a point mutation in a conserved tryptophan residue at amino acid 127 within the predicted Pbx interaction motif. (**B**) and (**C)** Immunoprecipitation analysis of HEK293 cells transfected with Tlx3, CBP and/or Pbx3 expression constructs. IP, immuno-precipitation; IB, immunoblotting. The lysate from cells transfected with an empty vector was used as a negative control. The expression levels of Tlx3-Myc, Myc-tagged mutants of Tlx3, FLAG-Pbx3, and CBP (input) are also shown.

To test the hypothesis that Tlx3 and CBP act cooperatively to promote glutamatergic neuronal differentiation, we first examined whether Tlx3 can bind CBP using co-immunoprecipitation assays. HEK293 cells were transiently cotransfected with Myc-tagged Tlx3 (Tlx3–Myc; Fig 1A) and CBP expression vectors. At 40 h after transfection, the total cell lysates were subjected to immunoprecipitation with anti-Myc antibody-conjugated beads, and the immunoprecipitate was then subjected to immunoblotting analyses with an anti-CBP antibody. In these conditions, Tlx3–Myc weakly co-immunoprecipitated with CBP (Fig 1B, lane 2 and Fig AA in S1 File, lane 2). To confirm this observation, we immunoprecipitated the cell extract with anti-CBP, which was followed by immunoblotting for Tlx3–Myc. However, Tlx3–Myc was not detected in the anti-CBP immunoprecipitates (Fig 1B, lane 2 and Fig AA in S1 File., lane 2), which again suggested only weak interactions between Tlx3 and CBP.

**Fig 2:**
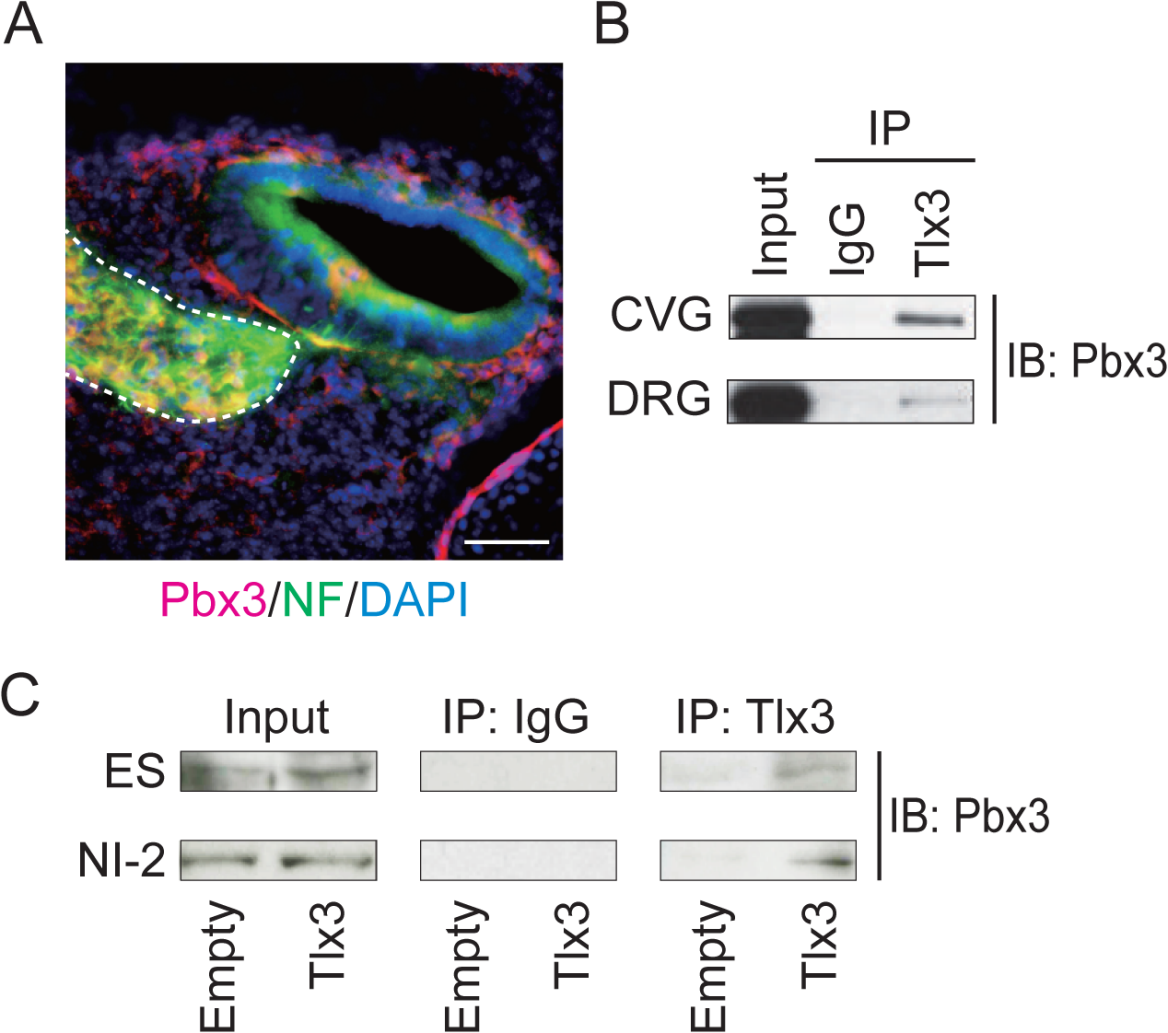
Pbx3 interacts with Tlx3 in embryonic sensory ganglia and neurons derived from Tlx3-expressing ES cells. (**A**) Pbx3 expression (red) in the inner ear of an E14 mouse embryo. The section was counterstained to detect the neuronal marker neurofilament (green) and with DAPI (blue). The region with the inner ear ganglia is marked by the dotted line. (**B**) Immunoprecipitation analysis of nuclear extracts from the cochleovestibular ganglion and dorsal root ganglion of E10 mouse embryos. Normal mouse IgG was used as a negative control. (**C**) ES cells were stably transfected with an empty vector (Empty) or Tlx3 expression vector (Tlx3). Nuclear extracts from undifferentiated ES (ES) cells and ES-derived cells at neural induction day 4 were subjected to immunoprecipitation (IP) with normal mouse IgG or an anti-Tlx3 antibody, and the resulting immunoprecipitates were subjected to immunoblotting (IB) using an anti-CBP antibody. The intracellular levels of Pbx3 (input) are also shown. ES, undifferentiated embryonic stem cells; NI, embryonic stem cells after neural induction. Scale bar: 50 μm.

These results led us to hypothesize that a cofactor required for Tlx3–CBP complex formation was missing in HEK293 cells. Hox proteins, such as Tlx3, are known to interact with Pbx cofactors, which are three amino acid loop extension-class homeodomain transcription factors, to control the transcription of their target genes [28–32]. Tlx3 possesses a Pbx interaction motif immediately upstream of the homeodomain. Furthermore, Pbx proteins bind directly to CBP [30]. In particular, pre-B-cell leukemia transcription factor 3 (Pbx3) was expressed in the developing spiral ganglion (Fig 2A), and *Pbx3*-mutant mice exhibit similar phenotypes to the defects seen in *Tlx3*-deficient mice. Furthermore, the immunoprecipitation analysis showed that Tlx3 and Pbx3 formed a protein–protein complex in developing sensory ganglia, such as the cochleovestibular and dorsal root ganglia of E10 mouse embryos (Fig 2B and Fig BA in S1 File). In addition, Tlx3 interacts with Pbx3 in neurally differentiated ES cells (Fig 2C and Fig BB in S1 File). Based on these observations, we speculated that Pbx3 might act as a scaffolding protein to support the assembly of Tlx3 and CBP, thereby facilitating their interactions. To test this possibility, HEK293 cells were triply transfected with Pbx3, Tlx3–Myc, and CBP expression vectors, and the cell extracts were subsequently subjected to immunoprecipitation with an anti-Myc antibody, followed by immunoblotting with an anti-CBP antibody. In this condition, Tlx3 co-immunoprecipitated with CBP to a greater degree than in cells expressing no Pbx3 (Fig 1C, lane 2 and Fig AB in S1 File, lane 2). Indeed, immunoprecipitation of the cell lysates with anti-CBP followed by immunoblotting with anti-Myc demonstrated substantial co-immunoprecipitation of Tlx3-Myc with CBP (Fig 1C, lane 2 and Fig AB in S1 File, lane 2). To determine whether Pbx3 overexpression influenced the immunoprecipitation efficiency of the antibodies used in this assay (rather than complex formation), we evaluated the amounts of Tlx3–Myc and CBP immunoprecipitation using their specific antibodies. Anti-Myc and anti-CBP appeared to pull down Tlx3–Myc and CBP, respectively, with a similar affinity, regardless of the presence or absence of Pbx3 (Fig 1B and 1C and Fig A in S1 File), thus excluding any potential confounding effects in the presence of Pbx3, and also indicating that Tlx3–CBP complex formation depends partly on Pbx3.

To determine the region(s) of the Tlx3 protein that binds CBP, we generated several Tlx3 mutants (Fig 1A): (i) a deletion mutant lacking the carboxyl-terminal region (amino acids 227–291; Tlx3ΔC); (ii) a deletion mutant lacking the homeodomain (amino acids 166–226; Tlx3ΔHD); and (iii) a point mutant in which an evolutionarily conserved tryptophan residue within the Pbx interaction motif, which is critical for the association between Hox11 and Pbx, was substituted with glycine (Tlx3-W127G). One of these Tlx3 mutants was transfected into HEK293 cells with the CBP and Pbx3 expression vectors, and whole cell extracts were subjected to immunoprecipitation followed by immunoblotting. Wild-type Tlx3 and Tlx3ΔC co-immunoprecipitated with CBP, whereas Tlx3ΔHD failed to co-immunoprecipitate with CBP (Fig 1B and Fig AA in S1 File). Because Pbx3 enhanced Tlx3–CBP binding (Fig 1B and 1C and Fig A in S1 File), we also tested whether the Pbx interaction motif is required for this association. However, Tlx3-W127G had no effect on the interaction between Tlx3 and CBP, regardless of the presence or absence of Pbx3 (Fig 1, lanes 2 and 5 and Fig A in S1 File, lanes 2 and 5).

### Tlx3 interacts with CBP in Tlx3-expressing ES-derived neurons

**Fig 3:**
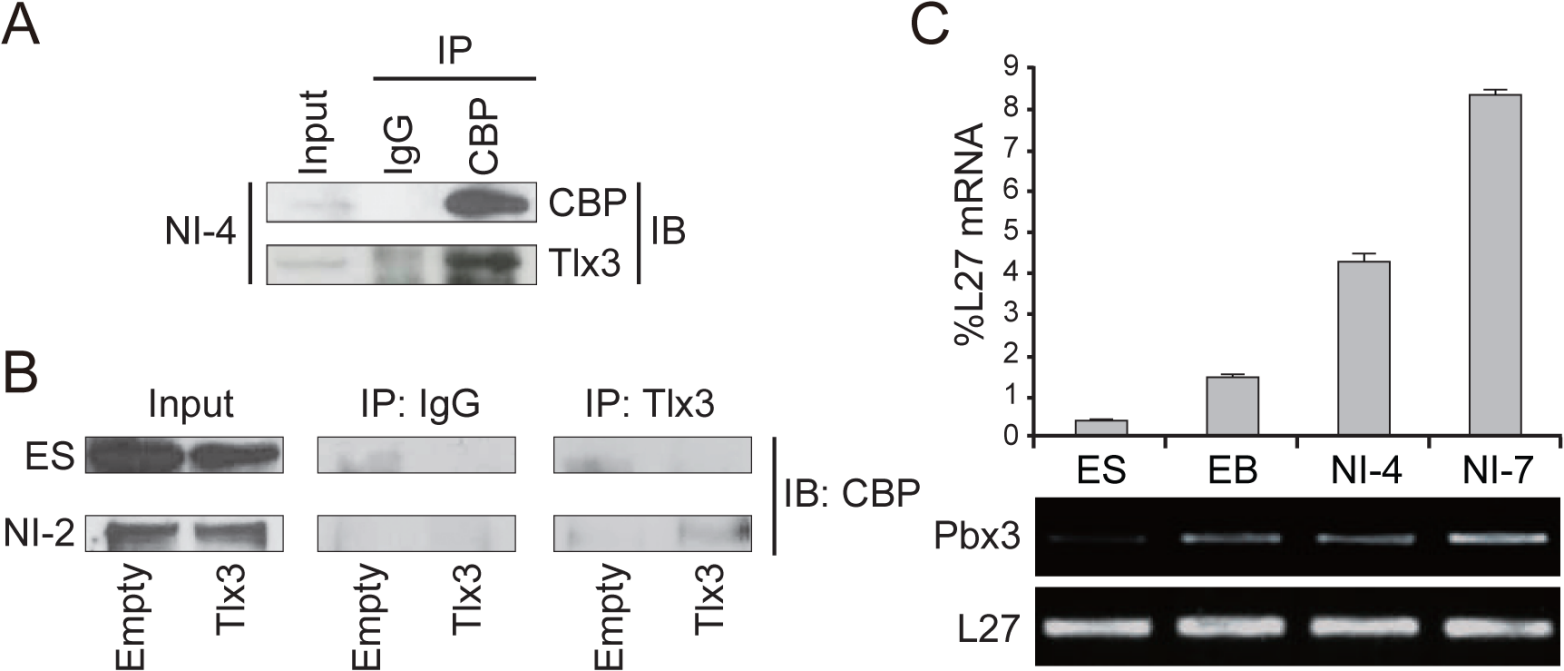
Tlx3 interacts with CBP only after ES cells differentiate into neurons. (**A**) Nuclear extracts from Tlx3-expressing ES-derived cells at neural induction day 4 were immunoprecipitated (IP) with normal mouse IgG or an anti-CBP antibody. The resulting immunoprecipitates were subjected to immunoblotting (IB) using either an anti-CBP or anti-Tlx3 antibody. The intracellular levels of Tlx3 and CBP (input) are also shown. **(B)** ES cells were stably transfected with an empty vector (Empty) or Tlx3 expression vector (Tlx3). Nuclear extracts from undifferentiated ES (ES) cells and ES-derived cells at neural induction day 2 were immunoprecipitated with normal mouse IgG or an anti-Tlx3 antibody. The resulting immunoprecipitates were subjected to immunoblotting using an anti-CBP antibody. The intracellular level of CBP (input) is also shown. (**C**) Quantitative RT-PCR analysis to evaluate changes in the *Pbx3* expression level in ES cells before and after neural induction. ES, undifferentiated embryonic stem cells; NI-4, ES-derived cells on day 4 after the start of neural induction.

To elucidate the physiological function of Tlx3–CBP binding, we used mouse ES cells stably expressing Tlx3. Using this model system, we previously demonstrated that the forced expression of Tlx3 is sufficient to induce ectopic expression of glutamatergic neuronal markers in mouse ES cells undergoing directed neural differentiation [4]. Moreover, the activity of excitatory postsynaptic currents was enhanced upon depolarization in neurons derived from Tlx3-expressing ES cells [4]. Accordingly, we chose Tlx3-expressing ES cells undergoing neural differentiation as a model system with which to recapitulate glutamatergic neuronal differentiation *in vitro*. To determine whether Tlx3 interacts with CBP in this system, Tlx3-expressing ES (Tlx3-ES) cells that had been directed to differentiate into neurons were harvested and subjected to co-immunoprecipitation assays. An anti-CBP antibody immunoprecipitated a Tlx3-immunoreactive band from the cell lysates of neurally induced Tlx3-ES cells, whereas it was undetectable when the anti-CBP antibody was replaced with nonspecific control IgG (Fig 3A and Fig CA in S1 File). In addition, co-immunoprecipitation of Tlx3 was detected in neurally differentiated Tlx3-ES cells, but not in undifferentiated ES cells (Fig 3B and Fig CB in S1 File).

Because Pbx3 enhanced co-immunoprecipitation of Tlx3 with CBP in HEK cells, we wondered if Pbx3 regulates the strength of the Tlx3–CBP interaction in ES cell-derived neurons. To explore this possibility, we examined changes in the Pbx3 expression level in ES cells undergoing neural differentiation. Quantitative PCR analysis showed that Pbx3 expression increased gradually during neural differentiation from ES cells (Fig 3C), suggesting that Pbx3 may indeed mediate differentiation-dependent interactions between Tlx3 and CBP.

### Tlx3 lacking the homeodomain is incapable of upregulating glutamatergic neurononal markers in ES-derived neurons

**Fig 4:**
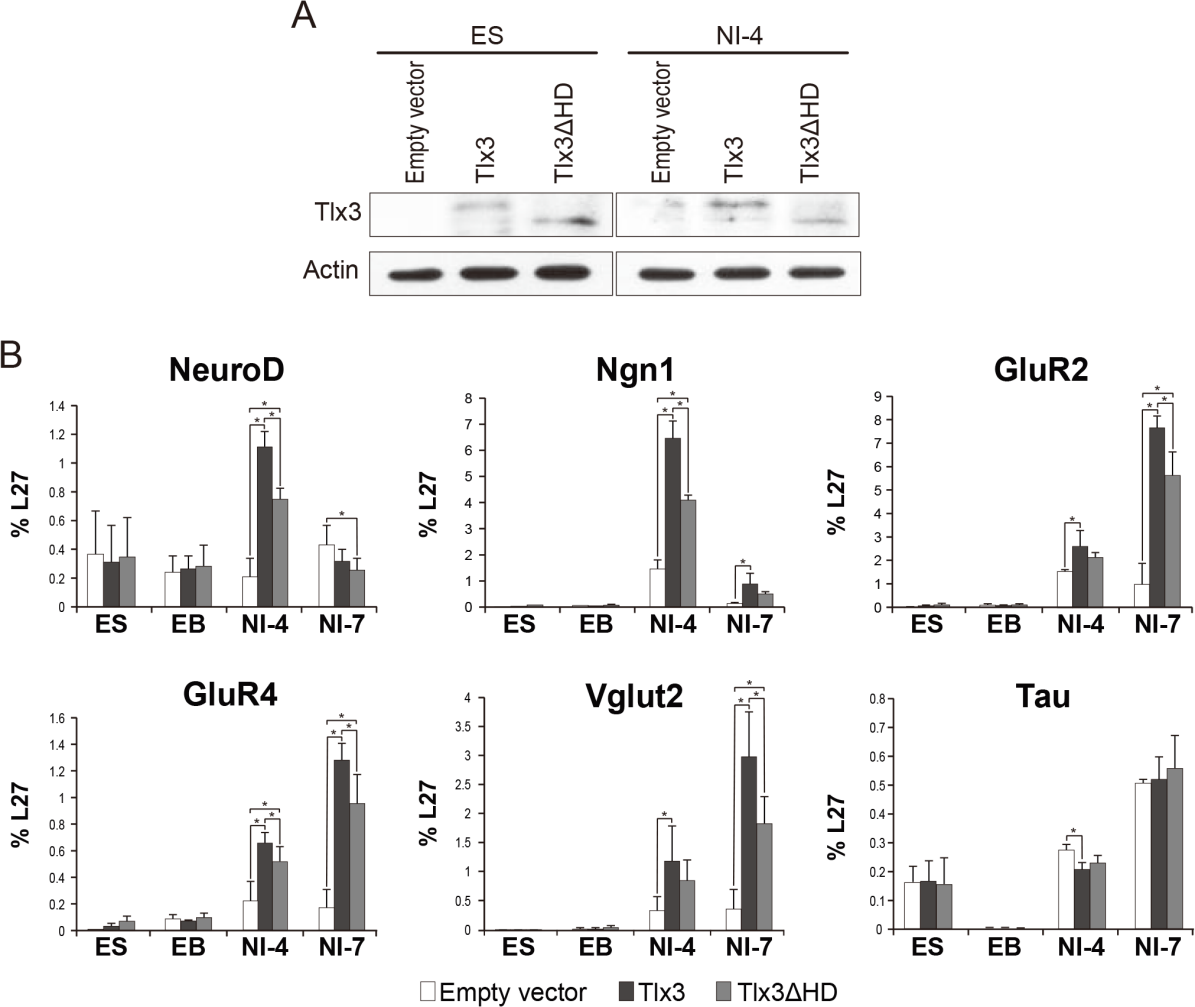
Effects of Tlx3 homeodomain deletion on glutamatergic neuronal marker gene expression in ES cell-derived neurons. (**A**) Confirmation of forced Tlx3 expression in ES cells stably expressing empty, Tlx3, or Tlx3ΔHD expression vectors. ES cells (ES) and ES cell-derived neurons (NI) were immunoblotted with an anti-Tlx3 antibody (upper panel). An anti-actin antibody was used as a loading control (lower panel). (**B**) Quantitative RT-PCR analysis for proneural markers (NeuroD, Ngn1), glutamate receptor subunits (GluR2, GluR4), the vesicular glutamate transporter (VGLUT2), and a pan-neuronal marker (Tau) in response to the forced expression of Tlx3 or Tlx3ΔHD in ES cells. White bars represent gene expression levels in empty vector expressing cells, dark gray bars in wild-type Tlx3-expressing cells, and light gray bars in Tlx3ΔHD-expressing cells. The expression levels were normalized against those of the housekeeping gene L27 (n = 3 culture plates; the data represent the average ± SD. *p < 0.05, one-way ANOVA). ES, undifferentiated embryonic stem cells; EBs, embryonic bodies; NI-4, ES-derived cells on day 4 after neural induction; NI-7, ES-derived cells on day 7 after the start of neural induction.

Next, we tested whether the homeodomain in Tlx3 is essential for Tlx3-mediated transcriptional activation of glutamatergic neuronal genes in ES cell-derived neurons. ES cells that stably expressed wild-type Tlx3, Tlx3ΔHD, or an empty vector (Fig 4A and Fig D in S1 File) were induced as previously described. Their differentiation states were assessed based on the expression of the proneural transcription factors NeuroD and Ngn1, the metabotropic glutamate receptors GluR2 and GluR4, and the glutamate transporter VGLUT2, because all these markers are upregulated by forced expression of Tlx3 [4]. Consistent with our previous study, the *NeuroD* and *Ngn1* mRNA levels were significantly higher on neural induction day 4 in Tlx3-expressing ES cell-derived neurons compared with those in ES cell-derived neurons lacking Tlx3, after which these genes were downregulated (Fig 4B). Moreover, the expression levels of *GluR2*, *GluR4*, and *VGLUT2* were significantly higher on neural induction day 4 in neurons derived from Tlx3-ES cells compared with those in ES cell-derived neurons lacking Tlx3, and the expression levels of these genes continued to increase until neural induction day 7. Thus, the presence of Tlx3 during induced differentiation appears to promote differentiation towards a glutamatergic neuronal subtype. The expression of the pan-neuronal marker *Tau* was also increased after neural induction. The expression levels of *Tau* were significantly lower on neural induction day 4 in neurons derived from Tlx3-ES cells compared with those in ES cell-derived neurons lacking Tlx3, but there was no significant difference in the expression level observed in the neurons derived from Tlx3-overexpressing ES cells vs. neurons derived from Tlx3 nonexpressing ES cells after neural induction day 7. The forced expression of Tlx3ΔHD led to similar temporal changes in the gene expression pattern in differentiated ES cells. However, the expression levels of *NeuroD*, *Ngn1*, *GluR2*, *GluR4*, and *VGLUT2* were significantly lower in neurons derived from Tlx3ΔHD-ES cells compared with those in neurons derived from wild-type Tlx3-ES cells. In striking contrast, there was no significant difference in the *Tau* expression level between neurons derived from Tlx3ΔHD-ES cells and those derived from wild-type Tlx3-ES cells.

**Fig 5:**
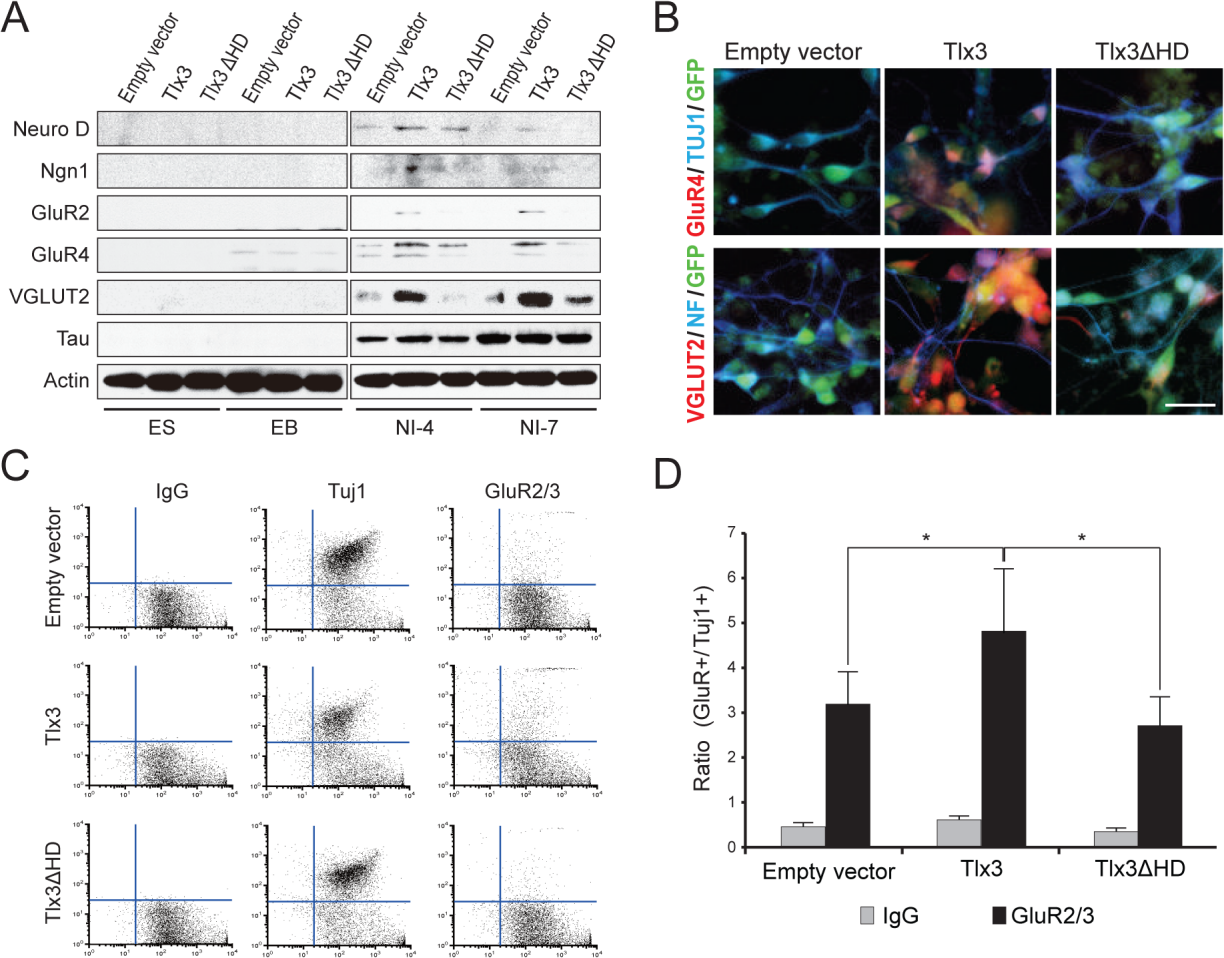
Effects of Tlx3 homeodomain deletion on glutamatergic neuronal marker proteins in ES-derived neurons. (**A**) Immunoblot analysis to evaluate the expression levels of glutamatergic neuronal marker proteins (NeuroD, Ngn1, GluR2, GluR4, and VGLUT2) and the pan-neuronal marker protein (Tau) in response to forced expression of Tlx3 or Tlx3ΔHD in ES cells. Whole lysates from ES cells stably transfected with the empty vector served as the negative control. Lysates from undifferentiated ES cells (ES), EBs, and ES-derived cells on day 4 (NI-4) or 7 (NI-7) after neural induction were analyzed by immunoblotting. As a control for protein loading, the blots were analyzed with antiactin antibodies (lower panel). **(B)** Immunocytochemical analysis of the expression of glutamate receptor subunits (GluR2 and GluR4; red) and pan-neuronal markers (Tuj1 and neurofilament; blue) in ES-derived cells at NI-7. Transgenic ES cells exhibited eGFP fluorescence (green). (**C**) Representative flow cytometric scatter plots of Tlx3- or Tlx3ΔHD-expressing ES-derived cells at NI-7. Cells stably transfected with an empty vector served as the negative control. Cells were stained with primary antibodies for glutamate receptor subunits (GluR2/3) or a pan-neuronal marker (Tuj1), followed by staining with AlexaFluor 568-conjugated secondary antibodies. The specificity of the primary antibodies was confirmed based on a comparison with the isotype control IgG. The fluorescence intensity of eGFP (marking transgenic ES cell-derived cells) is indicated on the *x*-axis and the fluorescence intensities of GluR2/3 and Tuj1 immunostaining on the *y*-axis. (**D**) Mean ratio of GluR2/3-positive relative to Tuj1-positive cells in GFP-positive cell populations. The values were normalized against Tuj1 (arbitrarily set to 1), and they are presented as the mean ± SD (*n* cells = 10,000, *p< 0.05, one-way ANOVA). ES, undifferentiated embryonic stem cells; NI-4, ES-derived cells at neural induction day 4; NI-7, ES-derived cells at neural induction day 7. Scale bar: 20μm.

To determine the corresponding protein expression levels, we performed immunoblot analyses using specific antibodies for NeuroD, Ngn1, GluR2, GluR4, VGLUT2, and Tau (Fig 5A and Fig E in S1 File). NeuroD, Ngn1, GluR2, and GluR4 proteins were detected in both Tlx3- and Tlx3ΔHD-ES cells grown in neural induction medium for 4 days but not in control ES cells. The expression levels of these proteins were also notably lower in Tlx3ΔHD-ES cell-derived neurons than those in Tlx3-ES cell-derived cells. After 7 days in neural induction medium, the expression levels of NeuroD and Ngn1 proteins were barely detectable in Tlx3-ES cell-derived neurons, whereas the expression levels of the GluR2 and GluR4 proteins remained high. However, the expression levels of GluR2 and GluR4 proteins in Tlx3ΔHD-ES cell-derived neurons were low, even at neural induction day 7. The VGLUT2 protein was barely detectable in Tlx3-ES cells at 4 days after the start of neural induction, whereas it was evident after 7 days. By contrast, expression of VGLUT2 in Tlx3ΔHD-ES cell-derived neurons was undetectable throughout induced differentiation. Consistent with the hypothesized function of Tlx3 as a glutamatergic differentiation driver rather than a general neural inducer, expression of the pan-neuronal marker Tau became detectable in all of the transgenic ES cell lines around neural induction day 4 and it was maintained until day 7. Immunofluorescent staining of GluR4 and VGLUT2 proteins was clearly detectable in Tlx3-ES cell-derived neurons but not in Tlx3ΔHD-ES cell-derived neurons (Fig 5B), consistent with the immunoblotting results.

To quantify the proportion of neurons expressing glutamate receptor subunits, we performed flow cytometry analyses for GluR2/3 and the pan-neuronal marker neuron-specific class III beta-tubulin (TUJ1) (Fig 5C and 5D). There were no significant differences among the proportions of TUJ1-positive cells derived from control ES cells, Tlx3-ES cells, or Tlx3ΔHD-ES cells, but the mean GluR2/3-positive to TUJ1-positive ratio was significantly higher in neurons derived from Tlx3-ES cells compared with those derived from control ES cells (Fig 5D). Moreover, Tlx3ΔHD-ES cell-derived neurons exhibited a significantly lower percentage of GluR2/3-positive cells than wild-type Tlx3-ES cell-derived neurons (Fig 5D).

### ES cell-derived neurons expressing mutated Tlx3 exhibit impaired Ca^2+^ homeostasis

**Fig 6:**
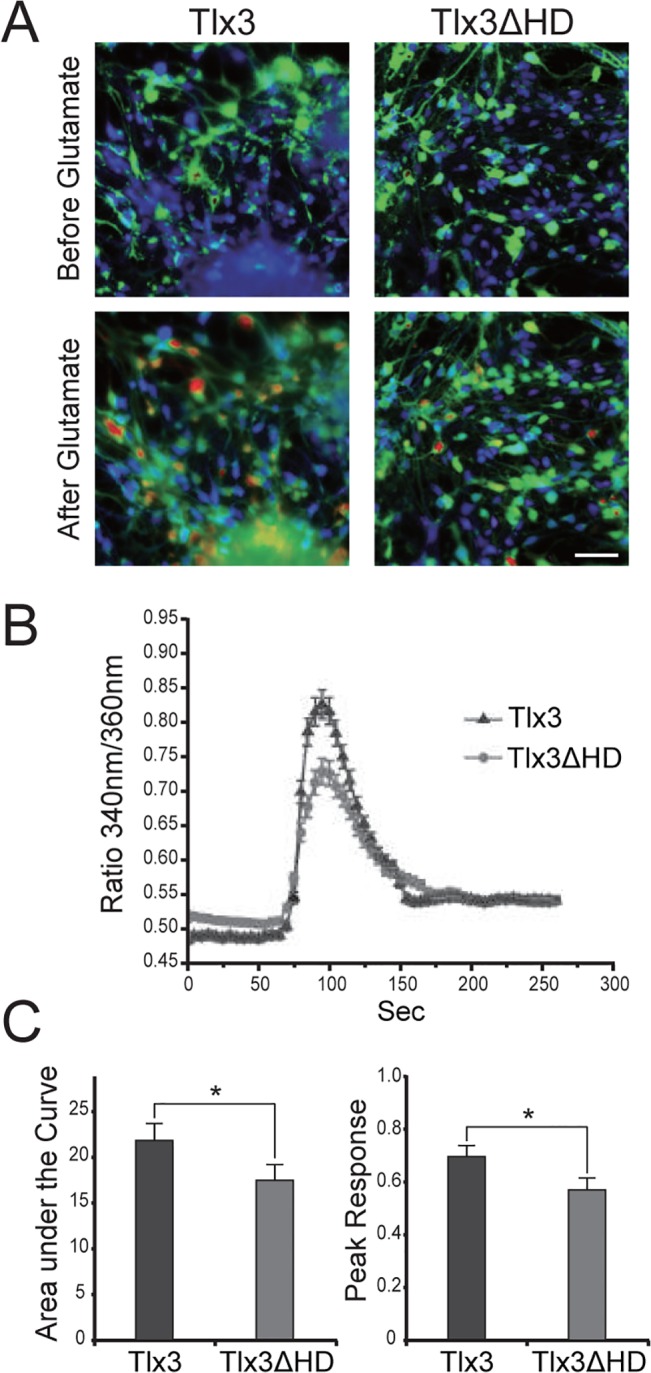
Effects of Tlx3 homeodomain deletion on the intracellular calcium in ES-derived neurons in response to glutamate stimulation. (**A**) Representative pseudocolor images of Fura-2-loaded ES-derived neurons transfected with Tlx3 (left panels) or Tlx3ΔHD (right panels) before (top rows) and after (bottom rows) exposure to 100 μM glutamate. These images are merged fluorescence images of Fura-2 (red, indicating intracellular calcium [Ca^2+^]_i_), GFP (green, denoting transgenic ES cell-derived cells), and DAPI (blue, marking living cells). (**B**) Time course of [Ca^2+^]_i_ changes in ES-derived neurons expressing Tlx3 (triangle) or Tlx3ΔHD (circle). The average response to 100 μM glutamate is shown. The error bars represent SDs. (**C**) Quantitative analysis of the [Ca^2+^]_i_ responses to 100 μM glutamate recorded in ES-derived neurons transfected with Tlx3 or Tlx3ΔHD. Bar graphs show the average area under the [Ca^2+^]_i_ curve and the peak [Ca^2+^]_i_ response to glutamate. Dark gray bars represent the [Ca^2+^]_i_ responses in Tlx3-expressing cells, and light gray bars represent the [Ca^2+^]_i_ responses in Tlx3ΔHD-expressing cells (*p< 0.05, *t*-test). Scale bar: 50 μm.

Activation of ionotropic glutamate receptors causes neural membrane depolarization and a concomitant influx of calcium through voltage-gated channels. Since our results revealed that deletion in the homeodomain of Tlx3 results in suppressed expression of the ionotropic glutamate receptor subunits GluR2/3 and GluR4, we wished to test whether Tlx3ΔHD leads to a decrease in the intracellular calcium concentration in response to glutamate (Fig 6A and 6B). Consistent with this hypothesis, intracellular calcium [Ca^2+^]_i_ imaging by Fura-2 microfluorometry detected a smaller glutamate-evoked peak [Ca^2+^]_i_ and area under the response curve ([Ca^2+^]_i_ × time) in Tlx3ΔHD ES cell-derived neurons when compared to those in Tlx3 ES cell-derived neurons (Fig 6C).

## Discussion

We previously reported that Tlx3 promotes selective upregulation of glutamatergic neuronal subtype genes in mouse ES cells undergoing neural differentiation, while suppressing genes associated with the GABAergic fate [4]. The present results have substantiated our previous study and demonstrated that (i) Tlx3 directly interacts with the transcriptional/epigenetic cofactor CBP; (ii) Tlx3 interacts with CBP through its homeodomain (amino acids 166–226); (iii) the interaction between Tlx3 and CBP is enhanced by Pbx3; (iv) the interaction between Tlx3 and CBP increases during neural differentiation from ES cells, which is accompanied with a gradual increase in Pbx3 in these cells; and (v) the deletion of the homeodomain in Tlx3 results in suppressed expression of glutamatergic neuronal phenotype markers and a reduction in the intracellular Ca^2+^ concentration in ES cell-derived neurons.

CBP and its close relative p300 act as global cofactors in transcription and epigenetic regulation by interacting with over 400 proteins [22, 23], suggesting that CBP/p300 is involved in a number of developmental pathways. For example, p300 interacts with two distinct transcription factors, STAT3 and Smad1, to promote astrocytic fate specification from neural stem cells [33]. A mouse genetics study showed that the transcription factor Ngn1 recruits CBP/p300 to a specific gene promoter that activates its transcription, thereby inducing neurogenesis [34]. Moreover, in the developing spinal cord, the interaction between CBP and the Ngn2/retinoic acid receptor complex is required for motor neuron specification [35]. The results of our present study further support the importance of the physical interaction between CBP/p300 and a transcription factor during neural development, and also strongly suggest a novel function of CBP in Tlx3-mediated neuronal subtype specification. Based on the established role for Tlx3 in glutamatergic neuronal specification [3]-[8] and the robust binding of Tlx3 to CBP in the presence of Pbx3 (Fig 1B) in HEK cells, we hypothesized that the Tlx3–CBP binding might have functional implications in pluripotent stem cells undergoing neural differentiation. Consistent with our hypothesis, we found that Tlx3 interacts with CBP in ES cell-derived neurons that stably express Tlx3 (Fig 3A and 3B). Furthermore, the expression levels of a subset of glutamatergic neuronal genes and proteins in ES cell-derived neurons, expressing Tlx3ΔHD with impaired CBP binding, was significantly lower than those in neurons expressing wild-type Tlx3 (Figs 4B and 5A). How CBP modulates the efficiency of Tlx3-mediated transcription, however, remains to be elucidated. It is possible that CBP might be recruited to the promoters and/or enhancers of glutamatergic genes via its interaction with Tlx3, thereby enhancing transcription by posttranslational modifications. In support for this, transcriptional activation by several Hox proteins has been shown to be mediated by the recruitment of CBP to gene promoters [30, 36]. In addition to its ability to act as a transcriptional activator, Tlx3 may function as a transcriptional repressor, depending on the developmental and cellular contexts. In fact, several other members of the Hox family proteins can inhibit the CBP HAT activity via a homeodomain-mediated interaction and the CBP-dependent transcriptional activity [37–39]. Tlx3 target genes are downregulated in Tlx3-expressing leukemic cells according to a genome-wide binding site analysis [40]. Tlx3 can also suppress specific GABAergic genes in neurons derived from Tlx3-expressing ES cells [4], so it is possible that Tlx3 acts as a glutamatergic/GABAergic selector by exerting binary transcriptional regulation, which could depend on epigenetic modifications and the recruitment of different cofactors to its DNA-binding loci. It is tempting to speculate that Tlx3 controls the balance of excitatory and inhibitory neurons via its opposing transcriptional activities.

**Fig 7:**
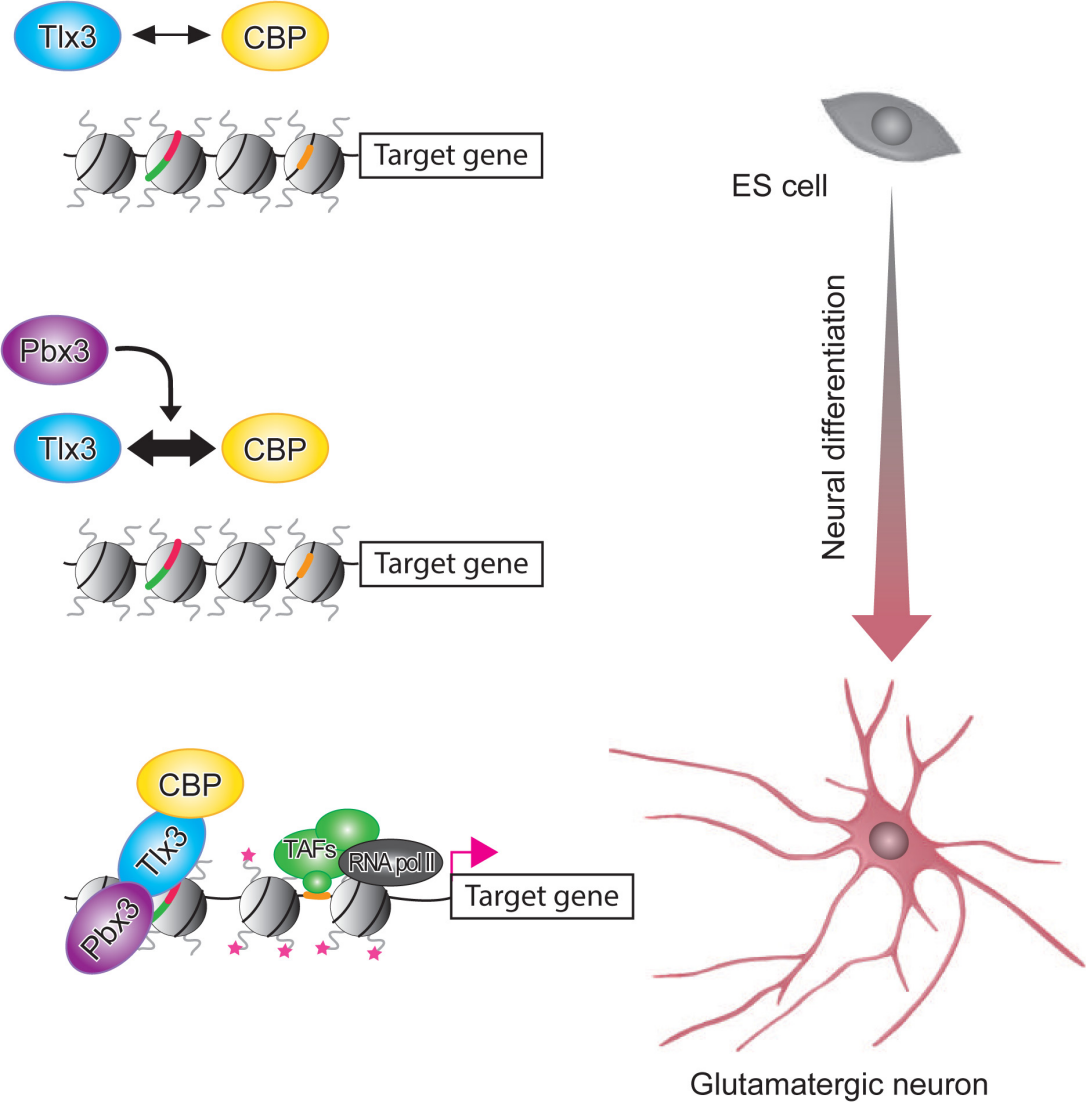
Schematic of the proposed mechanism underlying glutamatergic neuronal subtype specification mediated by Tlx3 and CBP. The black two-headed arrow denotes the interaction between Tlx3 and CBP. The thin two-headed arrow indicates a weak interaction, and the thick two-headed arrow denotes a stronger interaction. The black arrow denotes the enhancing effect of Pbx3 on the Tlx3–CBP interaction. The formation of the Tlx3–Pbx3–CBP complex leads to histone acetylation (red stars) of the promoters or enhancers of glutamatergic neuron-specific genes. The red arrow indicates transcriptional activation mediated by the Tlx3–Pbx3–CBP complex. Tlx3 (red) and Pbx3 (green) binding motifs in the promoter or enhancers of target genes are shown. The TATA box is also indicated (orange).

Forced expression of Tlx3 promotes expression of glutamatergic neuronal genes in ES cells after commitment to the neural lineage, whereas it has no effect in undifferentiated ES cells (Figs 4B and 5A). These results are consistent with our previous study [4], and demonstrate that Tlx3 is a context-dependent differentiation factor. A possible mechanism underlying this context dependency could involve the differentiation-dependent expression of Pbx cofactors. Pbx proteins enhance the DNA-binding specificity of Hox proteins (which themselves have low DNA-binding affinities), and are required for other aspects of Hox function [28–32]. In the present study, we obtained biochemical evidence that Pbx3 enhances the Tlx3–CBP interaction, and we found that Pbx3 expression gradually increases after the start of neural differentiation *in vitro* (Fig 3C). Moreover, the Tlx3–CBP interaction was present in neurally differentiated ES cells, but undetectable in undifferentiated ES cells (which expressed low levels of Pbx3; Fig 3B). Based on these results, we propose a model, by which Tlx3 promotes differentiation of ES cells towards a glutamatergic neuronal subtype in a context-dependent manner (Fig 7). According to this model, Tlx3 is unable to recruit CBP to its DNA-binding sites in undifferentiated ES cells due to a lack of constitutive Pbx3 expression. However, upon the initiation of neural differentiation, Pbx3 is upregulated and enhances the interaction between Tlx3 and CBP, which then triggers transcriptional activation of Tlx3 target genes.

Based on the currently available evidence, it is conceivable that cooperation between Tlx3 and Pbx3 triggers the recruitment of CBP to the promoters and/or enhancers, which leads to the acetylation of core histone proteins and enhanced activation of glutamatergic neuronal genes. Paradoxically, Tlx3 with a point mutation in the consensus Pbx3-binding site did not alter the degree of the interaction with CBP (Fig 1B and 1C), which suggests that direct Tlx3–Pbx3 binding is not required for the Tlx3–CBP interaction. However, Tlx3 only weakly co-immunoprecipitated with CBP in the absence of Pbx3 (Fig 1B). In addition, CBP has been reported to interact directly with Pbx [30], suggesting that Tlx3–Pbx3 complex formation bridged by CBP could facilitate the Pbx3-mediated enhancement of the Tlx3–CBP interaction. Hox proteins transcriptionally regulate target genes by binding at the gene promoter via their homeodomain [29]. In addition, Hox proteins can bind to target genes in the presence of Pbx family members in conditions where minimal or no binding is detected with Hox or Pbx alone [29]. In the present study, we found that Tlx3 co-immunoprecipitated with Pbx3 in Tlx3-expressing ES cells and their derived neurons (Fig 2C). These results indicate that the ΔHD mutation may diminish the cell fate selector function of Tlx3 by inhibiting its DNA-binding activity rather than its CBP interaction. Further investigations is required to determine the role of Pbx3 in the Tlx3-CBP interaction in order to gain a greater understanding of the context-dependent activity of Tlx3 as a master selector gene.

## Supporting Information

S1 File Full-scan immunoblotting images. Fig A. Images appeared in Fig 1B and 1C. (A) and (B) Immunoblotting with anti-Myc, anti-CBP, or anti-FLAG antibodies. Fig B. Images appeared in Fig 2B and 2C. (A) and (B) Immunoblotting with anti-Pbx3 antibody. Fig C. Images appeared in Fig 3A and 3B. (A) Immunoblotting with anti-CBP or anti-Tlx3 antibodies. (B) Immunoblotting with anti-CBP antibody. Fig D. Images appeared in Fig 4A. Immunoblotting with anti-Tlx3 or anti-actin antibodies. Fig E. Images appeared in Fig 5A. Immunoblotting with antibodies indicated on the right.
(DOCX)
