# Supplementary figures and images for: Tlx3 Promotes Glutamatergic Neuronal Subtype Specification through Direct Interactions with the Chromatin Modifier CBP
Source: PLoS One. 2015 Aug 10;10(8):e0135060. doi: 10.1371/journal.pone.0135060 (PMC4530954; doi:10.1371/journal.pone.0135060)

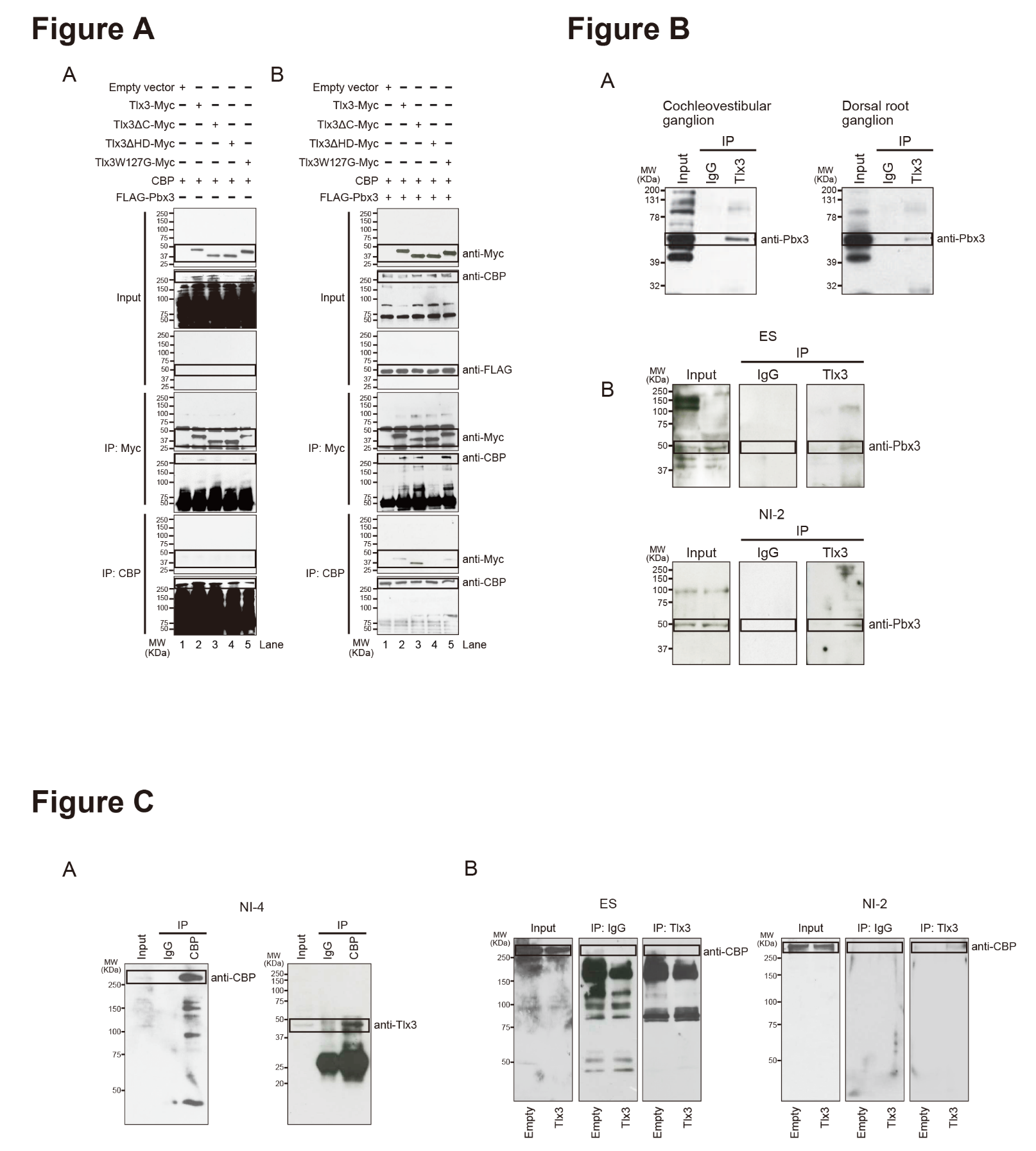


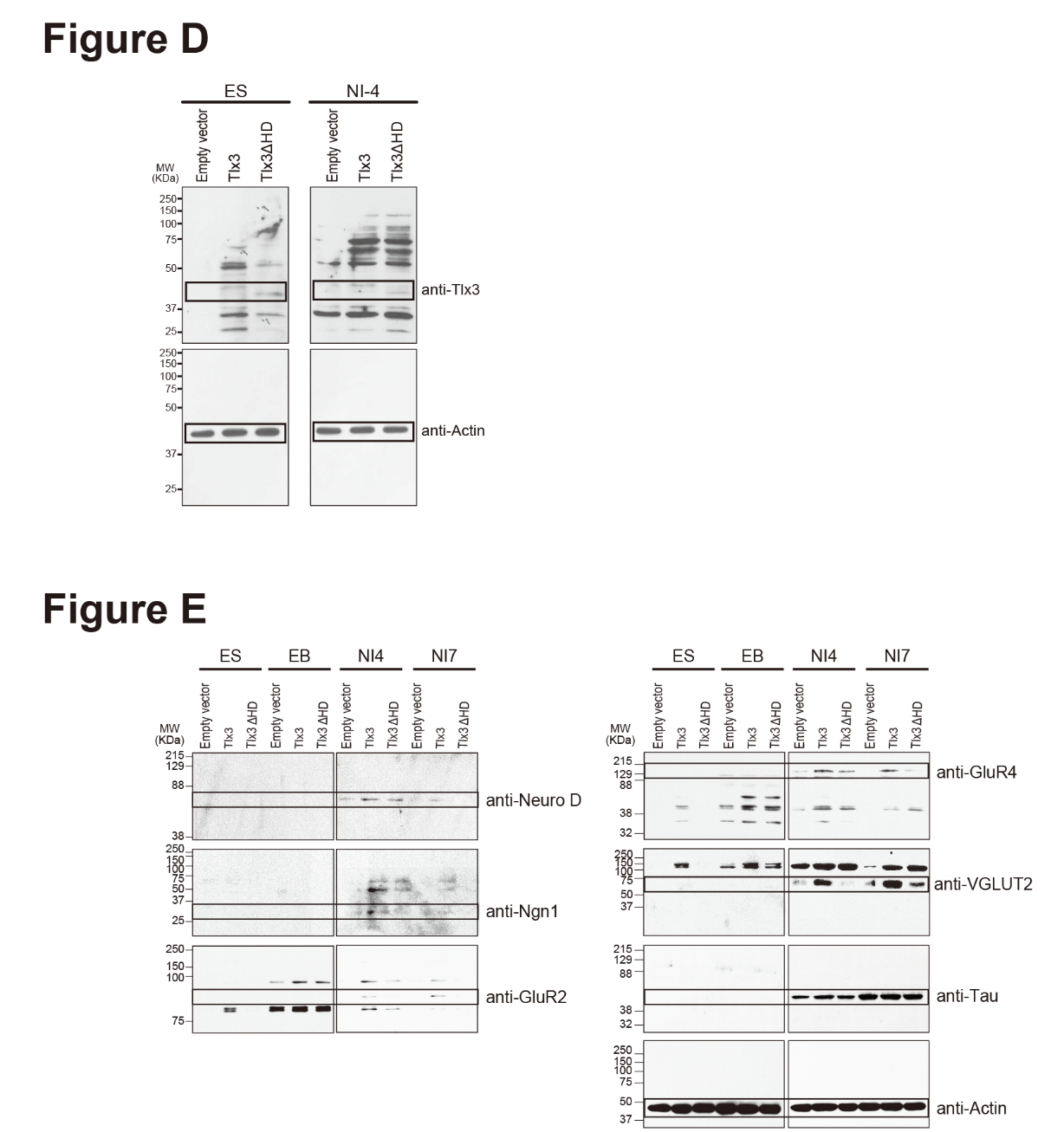

Supplement: S1 File — Fig A. Images appeared in Fig 1B and 1C. (A) and (B) Immunoblotting with anti-Myc, anti-CBP, or anti-FLAG antibodies. Fig B. Images appeared in Fig 2B and 2C. (A) and (B) Immunoblotting with anti-Pbx3 antibody. Fig C. Images appeared in Fig 3A and 3B. (A) Immunoblotting with anti-CBP or anti-Tlx3 antibodies. (B) Immunoblotting with anti-CBP antibody. Fig D. Images appeared in Fig 4A. Immunoblotting with anti-Tlx3 or anti-actin antibodies. Fig E. Images appeared in Fig 5A. Immunoblotting with antibodies indicated on the right. (DOCX) [file pone.0135060.s001.docx]
